# Novel environmental and sustainable approach for concurrent assay of antineoplastics in VMP regimen with a comprehensive Pharmacokinetic study

**DOI:** 10.1038/s41598-025-15078-6

**Published:** 2025-08-14

**Authors:** Marco M. Z. Sharkawi, Noha H. Amin, Norhan R. Mohamed, Shimaa S. Zaki, Loah R. Hemeda

**Affiliations:** 1 Pharmaceutical Analytical Chemistry Department, Faculty of Pharmacy , Beni-Suef University Alshaheed Shehata Ahmed Hegazy St. , Beni-Suef , 62514 Egypt; 2 Department of Medicinal Chemistry, Faculty of Pharmacy, Beni-Suef University, Beni-Suef, 62514 Egypt

**Keywords:** Bortezomib, Melphalan, Prednisolone, LC-MS/MS method, Green chemistry, Pharmacokinetics, Cancer, Medical research

## Abstract

Multiple myeloma (MM) is a blood cancer that, unfortunately, has a high morbidity and mortality rate. The VMP regimen, which includes bortezomib (BOR), melphalan (MEL), and prednisolone (PRD), is a safe and effective salvage regimen for refractory or relapsed MM. Up to now, there is no established analytical method to determine the VMP regimen, nor has any study investigated the kinetic interactions among its components, thereby highlighting the need for further clinical investigation. In light of this, an environmentally friendly, fast, sensitive, and precise LC-MS/MS method was established to determine bortezomib, melphalan, prednisolone, and sildenafil (an internal standard) simultaneously as part of the in vivo pharmacokinetics research carried out on rats. The established LC-MS/MS method was applied using a mobile phase composed of a mixture of methanol: 0.1% aqueous solution of formic acid and a ZORBAX Eclipse Plus C18 column (4.6 mm × 150 mm, 5 μm) as a stationary phase. The cited drugs were ionized through positive ionization and detected using multi-reaction monitoring (MRM) mode with the following precursor→product transitions: m/z 367.3→226.3 for BOR, m/z 305.0→168.2 for MEL, m/z 545.0→147.5 for PRD, and m/z 475.3→283.4 for SIL. Following FDA guidelines, the developed method was validated and showed acceptable ranges. Subsequently, it was employed in an in vivo investigation using rats, where the quantitative assessment of each drug was performed following both single and combined treatment. This allowed for the investigation of potential drug-drug interactions and the calculation of all pharmacokinetic parameters to monitor the therapeutic effects of those medications. To ensure the safety and environmental friendliness of the developed method, four assessment tools were applied: the assessment of green profile (AGP), blue applicability grade index (BAGI), analytical greenness metric for sample preparation (AGREEprep), and green analytical procedure index (GAPI).

## Introduction

**Fig. 1:**
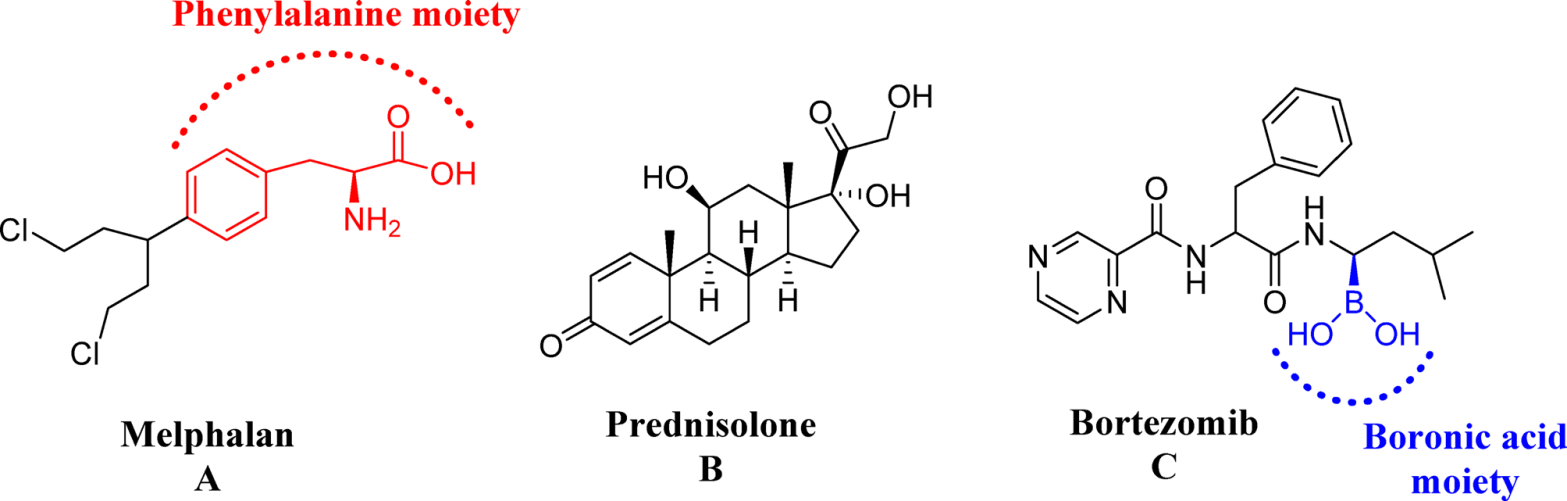
**A**–**C**. Chemical structures of three drugs involved in VMP regimen used by MM patients.

Globally, the incidence and prevalence of multiple myeloma (MM), the second most prevalent hematologic malignancy, are rising^1^. Nitrogen mustard alkylating agents like Melphalan (MEL) (Fig. 1A), derived from L-phenylalanine amino acid has been used for many years to treat MM by functioning as an immunostimulatory medication and a cytotoxic agent^2,3^. For a long time, MEL in conjunction with steroids like prednisolone (PRD) was the accepted course of treatment for MM patients, as PRD reduces the toxic effects of chemotherapy protocols in treating myeloma cells. (Fig. 1B)^4,5^.

The management of MM has evolved substantially over the past decades; however, it remains an incurable disease, and therapeutic advancements continue to be of paramount importance. Consequently, the era of novel-targeted therapy, in which medications and their combinations target specific pathways of tumor cell growth and survival, is replacing the conventional chemotherapy-oriented strategy against the highly proliferative cells. Bortezomib (BOR), a proteasome inhibitor (Fig. 1C), was the first drug containing modified dipeptidyl boronic acid to be approved by the Food and Drug Administration (FDA) and the European Medicines Agency (EMA) for refractory/relapsed MM, as it exhibits notable and substantial anti-myeloma action^6^.

The development of novel targeted agents is necessary to overcome resistance and improve outcomes. It has been demonstrated that the combination of bortezomib, melphalan, and prednisolone in the regimen known as the VMP is an effective, safe, and successful salvage regimen in patients with refractory or relapsed multiple myeloma^7,8^.

While clinical trials have affirmed the efficacy and safety of VMP in both newly diagnosed and relapsed/refractory MM patients, the pharmacokinetic interactions among these agents remain insufficiently studied. Frequent monitoring of antineoplastic drug levels and a solid understanding of pharmacokinetics (PKs) have become mandatory for ensuring the effective and safe use of chemotherapeutic regimens, with sensible dose adjustments based on studying the pharmacokinetic behaviour of each drug. Hence, there was a need for implementing rapid, sensitive, selective, and reproducible analytical methods for such routine clinical applications. Our group has recently developed many validated analytical methods for the therapeutic drug monitoring of various chemotherapeutic regimens^9,10^ followed by a full investigation study of the pharmacokinetic behaviours and the effect of each drug on the others in the same regimen protocol.

According to the reviewed literature, there are a number of analytical methods that can be used to determine BOR from various biological samples. These methods include high-performance liquid chromatography (HPLC)^11^ and liquid chromatography-tandem mass spectrometry (LC-MS/MS) method^12–14^. Additional analytical methods for determining MEL from biometrics have been found in the literature review. These include the thin-layer chromatography (TLC) method^15^, high-performance liquid chromatography (HPLC) method^16,17^, and liquid chromatography-tandem mass spectrometry (LC-MS/MS) method^18–20^.

On the other hand, PRD was analyzed from biometrics by the thin-layer chromatography (TLC) method^21^, HPLC method^22,23^, and the LC-MS/MS method^24,25^.

Based on the analytical methods revealed in the literature review, no analytical method has been established to determine binary or ternary combinations of the cited drugs. A novel and sustainable LC-MS/MS method was developed and validated for the simultaneous quantification of the studied drugs in the VMP combination chemotherapy with high sensitivity and selectivity without any interference between the cited drugs and the endogenous plasma constituents, followed by an in vivo study and an investigation of PK parameters after intraperitoneal administration in rats. In addition, researchers in the modern era are exerting a great deal of effort to develop novel and environmentally sustainable analytical methods. These methods include the substitution of potentially harmful solvents with safer alternatives, the reduction of pretreatment steps, and the restriction of sample preparation, energy consumption, and solvent consumption. The ecological friendliness of the suggested methods was assessed in our study using a variety of tools, including the assessment of green profile (AGP)^26^, blue applicability grade index (BAGI)^27^, analytical greenness metric for sample preparation (AGREEprep)^28^, and green analytical procedure index (GAPI)^29^.

## Experimental

### Instruments

The separation was accomplished with the help of an Agilent 1260 Infinity HPLC system equipped with a quaternary pump and a ZORBAX Eclipse Plus C18 column (4.60 mm × 150 mm, 5 μm) as the stationary phase. Mass detection was performed using an Agilent 6460 Triple Quadrupole mass spectrometer (Agilent Technologies, USA) equipped with an electrospray ionization source, and managed by the Agilent MassHunter Workstation software − Data Acquisition (ver. B.07.00). https://www.agilent.com/Library/usermanuals/Public/G3335-90166_QQQ_Concepts.pdf.

A Vortex mixer 300 VM, manufactured by Gemmy Industrial Corporation in Taiwan was employed for thorough sample mixing, while an electrical centrifuge (Hettich, Germany) that operates from − 20 to + 40 °C with a speed range of 800 − 15,000 rpm was utilized for sample centrifugation, featuring a capacity of 32 × 15 mL and operating at a voltage range of 200 to 240 V.

### Materials and reagents

#### Pure samples

BOR, MEL, and PRD were provided from Sigma-Aldrich CO., Darmstadt, Germany, which was purchased through the Egyptian International Center for import, Cairo, Egypt. Based on the reported methods, their purity levels were determined to be between 99.13% and 102.75%. The internal standard (Sildenafil) was kindly provided by EIPICO CO., El Horria St., Zone B ‏‎10th of Ramadan City, Egypt, having a purity level of 99.32%, as indicated by the manufacturer’s certificate of analysis.

#### Chemicals and solvents

The HPLC grade of methanol and formic acid were provided from Sigma-Aldrich CO., Darmstadt, Germany, which was supplied through Egyptian International Center for Import, Cairo, Egypt. Deionized water was obtained from SEDICO Pharmaceuticals Co., 6th October City, Egypt.

### Chromatographic conditions

The studied drugs were analyzed using analytical column C_18_ (4.60 mm × 150 mm, 5 μm) and a mixture of methanol: 0.1% aqueous solution of formic acid (gradient elution) at 1.3 mL/min flow rate as developing system. Furthermore, mass spectrometric conditions were set at: capillary voltage (3500 v), gas temperature (325 °C), spray gas pressure (400 Pa), nebulizer pressure (40 psi), and dwell time (200 ms). Cone voltages used for detection were 180 for BOR, 20 for MEL, 31 for PRD, and 110 for SIL, while collision energies were found to be 20, 35, 14, and 45 for BOR, MEL, PRD, and SIL, respectively. The electrospray ionization (ESI) source was operated on the positive ion detection mode and detection of the ions was carried out in the multiple-reaction monitoring mode (MRM).

### Prepared solutions

#### Stock standard solutions

Stock solutions of BOR, MEL, PRD, and SIL (1000 µg/mL) were separately prepared in four volumetric flasks with a volume of 10-mL using a solvent of methanol HPLC.

#### Working standard solutions

Two working standard solutions (10 µg/mL) and (1 µg/mL) for each of BOR, MEL, PRD while of 10 µg/mL for IS (SIL) were prepared by performing a separate transfer of equivalent volumes from their corresponding stock solutions into seven volumetric flasks with a volume of 10-mL and adding methanol until the volume reaches the mark to get the required concentration.

### Procedure

#### Construction of calibration curves

Various samples within the concentration range of 0.0005–10 µg/mL for BOR, MEL, and PRD were prepared into three separate sets of 5-mL volumetric flasks using their previously prepared standard solutions. Subsequently, SIL (0.25 mL) was added from its corresponding working solution (10 µg/mL) to each flask, followed by the addition of drug-free rat plasma (0.5 mL), and the volume was filled up with methanol. Following that, the vortex was used for two min to mix the prepared solutions, and centrifugation was achieved for ten minutes at 4000 rpm and 4 °C to remove the precipitated plasma and obtain the clear supernatant, which was separated and automatically injected (10 µL). Moreover, calibration curves were constructed for the cited components, relating the determined peak-area ratios (concerning the internal standard) to their respective concentrations, and equations of linear regression were computed.

#### Quality control samples (QCs)

In order to cover the different ranges of QC samples, various concentrations of BOR, MEL, and PRD were prepared: the lower limit of quantification (LLOQ), low-quality control (LQC), middle-quality control (MQC), and high-quality control (HQC), which were respectively, 0.0005, 0.0015, 0.1, and 1 µg/mL for each drug. All the prepared samples were directly stored till the time of analysis at − 20 °C.

#### Treatment of animals and collection of plasma samples

All animal-related procedures were conducted in accordance with the Guide for the Care and Use of Laboratory Animals^30^. The study was approved by the Institutional Animal Care and Use Committee of Beni-Suef University (Ethics Committee for Animal Experimentation) under approval number 024–036.

Forty-nine adult male Wistar rats, weighing between 150 and 250 g, obtained from the Animal House of Nahda University in Beni-Suef (NUB), Egypt, were randomly assigned to seven groups (n = seven per group). Each group was housed for 6 days under standard laboratory conditions, including a 12 to 14-hour light cycle and a temperature range of 22 to 25 °C, in a well-ventilated environment. Group I served as the control group, which received saline only and did not get any medication. Groups II-VII received various treatments: group II was administered a dose of 0.25 mg/kg of BOR, group III received 2 mg/kg of MEL, group IV was given 10 mg/kg of PRD, group V received a combination of 0.25 mg/kg of BOR and 10 mg/kg of PRD, group VI was treated with a combination of 2 mg/kg of MEL and 10 mg/kg of PRD, and finally, group VII was given a combination of 0.25 mg/kg of BOR, 2 mg/kg of MEL, and 10 mg/kg of PRD. Doses were calculated and prepared in saline in concentration of 0.2 mg/mL for BOR, 1 mg/mL for MEL, and 5 mg/mL for PRD and then injected intraperitoneally.

Following the administration of the drug, a blood sample of 0.5 mL was collected from the orbital plexus of the rats at various time intervals (0.25, 0.5, 1, 3, 6, 8, and 24 h). The blood sample was then collected in heparinized tubes and subjected to centrifugation (4000 rpm) for twenty minutes to separate the plasma from the blood sample. The plasma was kept at − 20 °C till the time of analysis.

At the analysis time, 0.5 mL of each plasma sample was taken after being thawed at room temperature, and 50 µL of IS was added from its corresponding working solution (10 µg/mL). Following that, methanol was added until the volume reached 1 mL, then mixed by vortex for 2 min, and centrifuged at 4000 rpm for 10 min. The clear supernatant was separated and analyzed using the same previous procedure. The plasma concentration of each drug administered in each group was then calculated, using the computed equations, and plotted versus the withdrawal time interval.

### Application to PK study

The plasma concentrations of BOR, MEL, and PRD were assessed and compared when administered individually and in combinations, subsequently establishing a relationship concerning their time intervals. PK parameters were investigated: half-life (t_1/2_), apparent volume of distribution (Vd), clearance (Cl), and elimination rate constant (k). Furthermore, the difference between groups was tested using the Analysis of Variance (ANOVA) test.

## Results and discussion

An innovative, environmentally friendly, and highly accurate LC-MS/MS method was developed for this study to investigate and determine the impact of BOR, MEL, and PRD on plasma levels of each other through in vivo research.

### Method optimization

A validated LC-MS/MS method was established to provide a green, reliable, and rapid method for simultaneously determining BOR, MEL, and PRD. Different chromatographic conditions such as the mobile phase composition and flow rate, were first tested to obtain efficient and good separation with high sensitivity and selectivity. Initially, a mixture of methanol and water in different ratios (50:50, 70:30, 30:70 80:20, and 90:10) were tested. However, complete separation with a reasonable time between the cited drugs could not be achieved in addition emerging of asymmetric peaks with poor tailing factor using this kind of solvent or elution technique. So gradient elution was employed to attain an appropriate polarity condition for each drug. Moreover, the tailing peaks were improved through PH modification using formic acid, as indicated in the literature survey^14,20,24^.

**Fig. 2:**
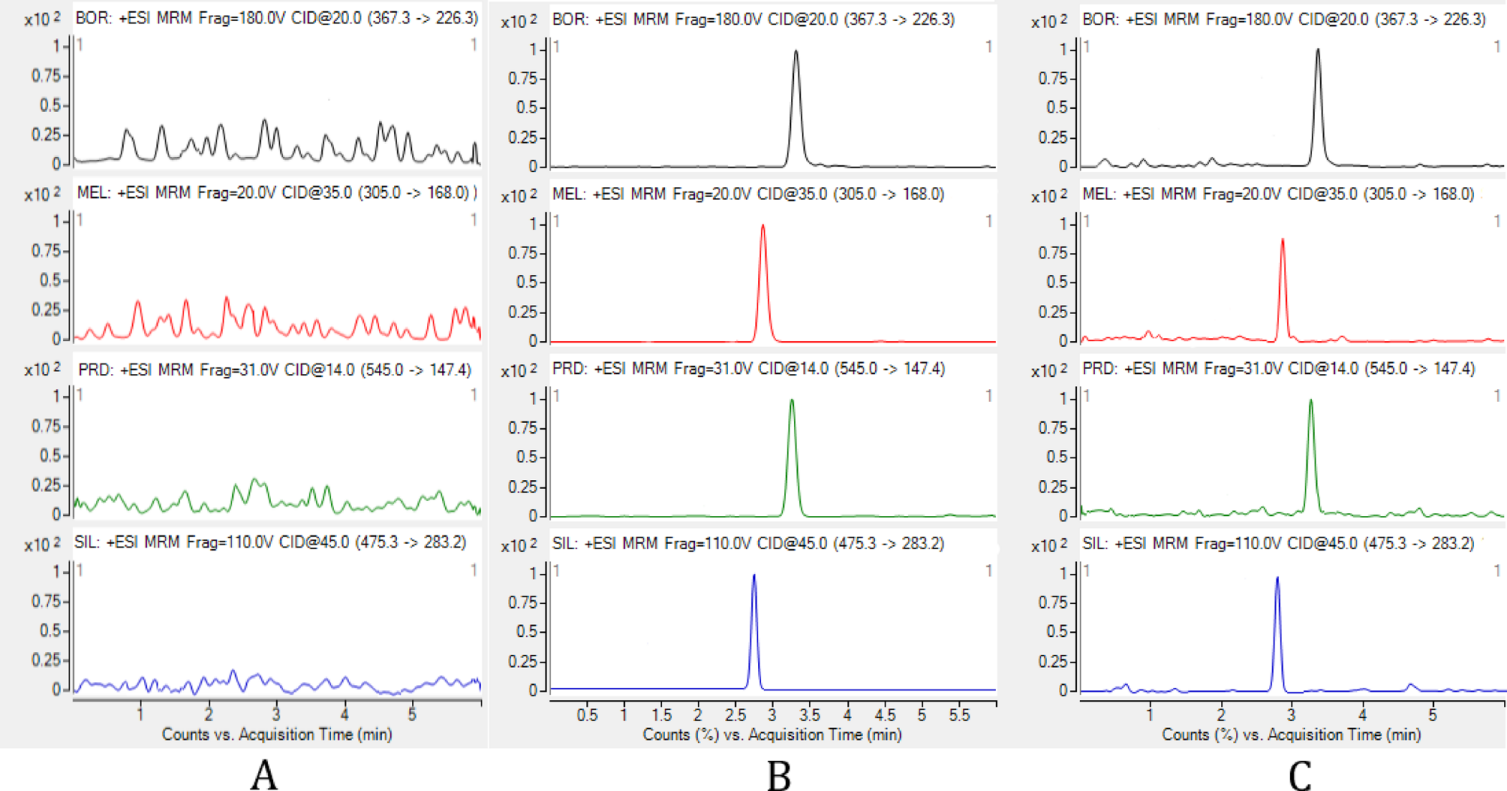
**A**–**C**. LC-MS/MS chromatograms of blank plasma (**A**), blank plasma spiked with 0.0005 of each BOR, MEL, and PRD using 0.5 µg/mL of SIL (**B**), and rat plasma sample collected from a rat after 0.5 h (**C**).

**Fig. 3:**
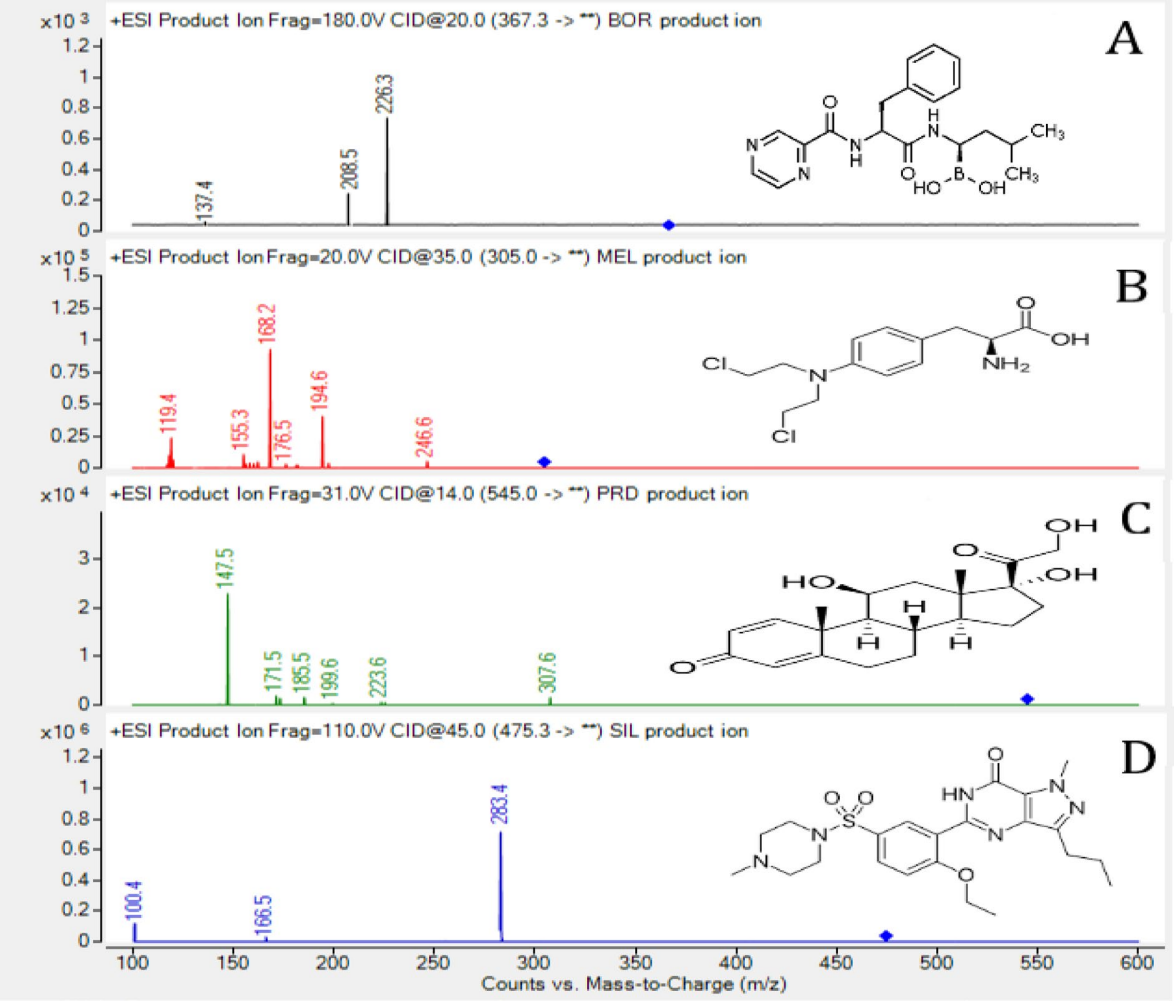
**A**–**D**. Product ion mass spectra of bortezomib (**A**), melphalan (**B**), prednisolone (**C**), and sildenafil (**D**).

Consequently, mixture of methanol: 0.1% aqueous solution of formic acid with the ratio of (40:60) at 1.3 mL/min flow rate, was the starting point for about half a minuet was very critical to assure the rapid elution and good resolution of SIL and MEL, then changing the ratio to (80:20) that suited other drugs elution at reasonable time with acceptable symmetric peaks, all the gradient programming details were summarized in Table 1. Following that chromatographic conditions the cited drugs were eluted at retention time of 2.75, 2.86, 3.21, and 3.30 min for SIL, MEL, PRD, and BOR, respectively (Fig. 2A-C). The second phase includes the optimization of mass spectrometric settings by analyzing the studied drugs separately to provide the optimal forms for the resolved peaks. For all drugs, the electrospray ionization (ESI) source was set to the positive ion detection. Moreover, other parameters such as capillary voltage, collision energy, and cone voltage were optimized to obtain the best sensitivity. The mass transition was m/z 367.3→226.3 for BOR, m/z 305.0→168.2 for MEL, m/z 545.0→ 147.5 for PRD and m/z 475.3→283.4 for SIL (Fig. 3A-D).

**Table 1:** Gradient elution technique of the mobile phase utilized in the LC-MS/MS method for the concurrent quantification of bortezomib, melphalan, and prednisolone.

Time (min)	Methanol %	0.1% aqueous solution of formic acid	Flow rate (ml/min)
0 ~ 0.5	40	60	1.3
0.5 ~ 4.5	80	20	1.3
4.5 ~ 6.0	40	60	1.3

### Method validation

The FDA guidelines’ recommendations were followed to validate the established LC-MS/MS method^31^.

#### Linearity of calibration curves

The ranges of linear calibration curves were found to be 0.0005–10 µg/mL of each of BOR, MEL, and PRD. The lower limit of quantification (LLOQ), where the analyte response should be ≥ five times the analyte response of the zero calibrator, was 0.0005 µg/mL while, the upper limit of quantification (ULOQ) was 10 µg/mL. All regression parameters were presented in Table 2.

#### Accuracy and precision

Testing the accuracy and precision of the developed LC-MS/MS method was achieved by analysis of quality control samples. A triplicate analysis was performed on the prepared samples on the same day to test intra-day accuracy and precision. However, the analysis of the same samples was performed on three consecutive days to test inter-day accuracy and precision. Intra and inter-day precision of the LC-MS/MS method was confirmed by acceptable values of % RSD within the range of 1.54 to 5.55% for BOR, 2.22 to 5.82% for MEL, and 1.08 to 6.04% for PRD. On the other hand, the agreeable % recoveries (100 ± 15%) of the tested samples assured the accuracy of the established method, as indicated in Table 3.

**Table 2:** Regression and statistical parameters of the developed LC-MS/MS method for determination of bortezomib, melphalan, and prednisolone.

Parameters	BOR	MEL	PRD
Range	0.0005-10 µg/mL	0.0005-10 µg/mL	0.0005-10 µg/mL
LLOQ	0.0005 µg/mL	0.0005 µg/mL	0.0005 µg/mL
Intercept	0.0006	0.0078	0.0053
Slope	0.3343	0.6476	1.0451
Correlation Coefficient	0.9999	0.9999	0.9999

**Table 3:** Intra-day and inter-day accuracy and precision for determination of bortezomib, melphalan, and prednisolone in rat plasma by the developed LC-MS/MS method. * Average of five determinations.$\text{**Bias}=\:\frac{(\text{m}\text{e}\text{a}\text{s}\text{u}\text{r}\text{e}\text{d}\:\text{c}\text{o}\text{n}\text{c}\text{e}\text{n}\text{t}\text{r}\text{a}\text{t}\text{i}\text{o}\text{n}-\text{t}\text{h}\text{e}\text{o}\text{r}\text{t}\text{i}\text{c}\text{a}\text{l}\:\text{c}\text{o}\text{n}\text{c}\text{e}\text{n}\text{t}\text{r}\text{a}\text{t}\text{i}\text{o}\text{n})}{\text{t}\text{h}\text{e}\text{o}\text{r}\text{t}\text{i}\text{c}\text{a}\text{l}\:\text{c}\text{o}\text{n}\text{c}\text{e}\text{n}\text{t}\text{r}\text{a}\text{t}\text{i}\text{o}\text{n}}$

Analyte	Concentration added µg/mL	Intra-day		Inter-day	
		Mean recovery% ± RSD^*^	Bias (%) ^**^	Mean recovery% ± RSD^*^	Bias (%) ^**^
BOR	0.0005 0.0015 0.1 1	93.70 ± 4.62 106.19 ± 2.69 98.68 ± 5.55 102.18 ± 3.05	−6.30 6.19 −1.32 2.18	104.72 ± 1.54 90.75 ± 5.41 96.20 ± 2.37 89.47 ± 2.65	4.72 −9.25 −3.80 −10.53
MEL	0.0005 0.0015 0.1 1	102.57 ± 5.20 111.94 ± 3.88 103.11 ± 3.15 95.81 ± 2.22	2.57 11.94 3.11 4.19	91.37 ± 2.42 88.04 ± 3.40 93.26 ± 5.82 102.85 ± 3.10	−8.63 −11.96 −6.74 2.85
PRD	0.0005 0.0015 0.1 1	104.28 ± 1.86 92.63 ± 3.09 107.19 ± 3.25 102.07 ± 4.67	4.28 −7.37 7.19 2.07	101.70 ± 1.08 94.97 ± 6.04 105.23 ± 1.94 103.74 ± 2.92	1.70 −5.03 5.23 3.74

* Average of five determinations.

#### Selectivity

To assure the selectivity of the method, blank plasma samples from seven different individual sources (group I, *n* = 7) was compared to plasma spiked at LLOQ concentration levels of each BOR, MEL, PRD, and SIL, which revealed that there was no significant interference between the cited drugs and the endogenous plasma constituents (Fig. 2A-C).

#### Extraction recovery and matrix effect

Extraction recovery of BOR, MEL, PRD, and SIL was estimated by comparing percentage recoveries of pure quality control samples to other plasma samples that were spiked with the same drug concentrations of the cited drugs in seven different plasma sources. The results showed that the developed method was effective in extracting the drugs without interference from the plasma matrix (100 ± 15%), as shown in Table 4.

**Table 4:** Recovery results for determination of bortezomib, melphalan, and prednisolone in rat plasma by the developed LC-MS/MS method.

Analyte	Concentration added µg/mL	Mean recovery% ± RSD^*^
BOR	0.0015 0.1 1	92.52 ± 5.94 109.85 ± 3.72 89.66 ± 6.12
MEL	0.0015 0.1 1	105.38 ± 2.16 98.72 ± 4.81 91.53 ± 3.03
PRD	0.0015 0.1 1	104.90 ± 4.79 97.32 ± 2.12 106.01 ± 4.33

* Average of five determinations.

#### Stability

The cited compounds’ stability was assured after exposing quality control samples to various conditions, including 6 h at room temperature (25 °C) for Bench-Top stability, 24 h in the auto-sampler for Post Preparative stability, and three freeze-thaw cycles [Freeze and thaw stability] through thawing from − 20 °C to room temperature. The acceptable range of the % recoveries (100 ± 15%) confirmed the stability of the method, as indicated in Table 5.

**Table 5:** Stability results of bortezomib, melphalan, and prednisolone in plasma under different conditions by the developed LC-MS/MS method.

Analyte	Concentration added µg/mL	% Remaining^*^		
		Room temperature for 6 h	Auto-sampler for 24 h	Three freeze-thaw cycle
BOR	0.0015 0.1 1	98.04 96.25 111.71	107.50 102.36 95.18	94.49 101.61 96.03
MEL	0.0015 0.1 1	94.30 101.92 105.57	86.13 88.06 91.22	102.39 90.97 94.56
PRD	0.0015 0.1 1	106.23 92.77 103.64	97.45 103.52 90.38	105.94 93.28 106.12

*Average of five determinations.

#### Carryover

In order to test carryover, a blank methanol solution was injected following the highest concentration calibration standard. Before and after each run, blank methanol was injected multiple times to wash the injection site and syringe, confirming that no carryover was observed throughout the analysis.

### Assessment of the greenness of the established method

An important principle of “green chemistry” is the consideration of potential human and environmental impacts of the analytical procedures. It is crucial to determine whether analytical methods are environmentally friendly with less toxic effects on both people and the environment. In this investigation, the developed LC/MS/MS method was evaluated using the following different metric approaches, which confirmed its superiority over the other reported methods regarding the use of greener solvents and waste consumption, as the reported methods have used acetonitrile and a higher volume of mobile phase^11,12,18,19,25^.

#### Assessment of green profile (AGP)

**Fig. 4:**
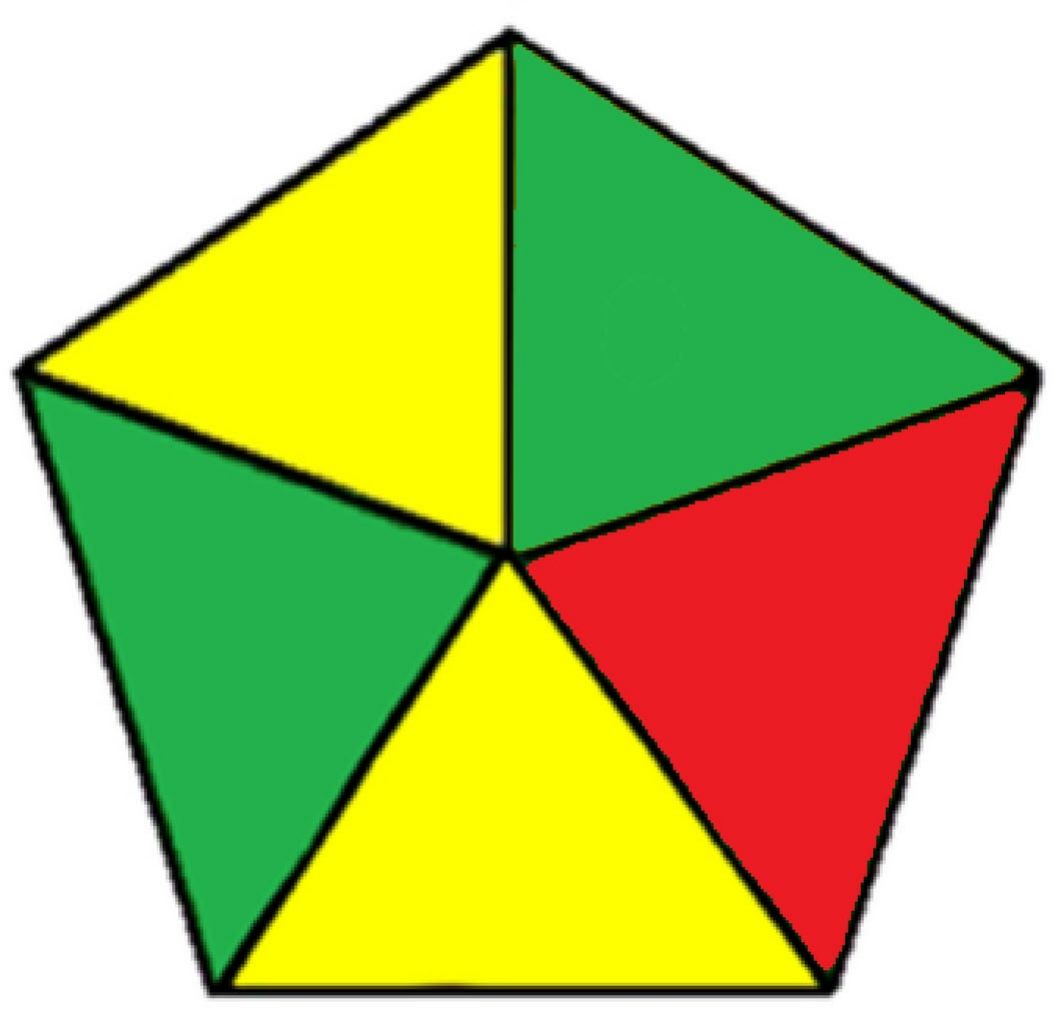
Assessment of Green Profile (AGP)-colored pictogram of the developed LC-MS/MS method.

This tool is semi-quantitative and descriptive, with simplicity in method selection and parameter comparison^26^. To evaluate the analytical process’s eco-friendliness, the AGP is structured into five parts that deal with health, safety, the environment, energy, and waste. The pictogram uses three colors to illustrate National Fire Protection Association (NFPA) scores to determine each section’s green rating of the developed method as presented in (Fig. 4).

#### Blue applicability grade index (BAGI)

This method^27^ focuses on the uses of White Analytical Chemistry and can be seen as a complement to established green metrics. It includes ten main criteria to assess the methods’ eco-friendliness, such as the type of analysis, the hourly sample throughput, the required instrumentation, the ability for simultaneous sample treatment, and the level of automation.

**Fig. 5:**
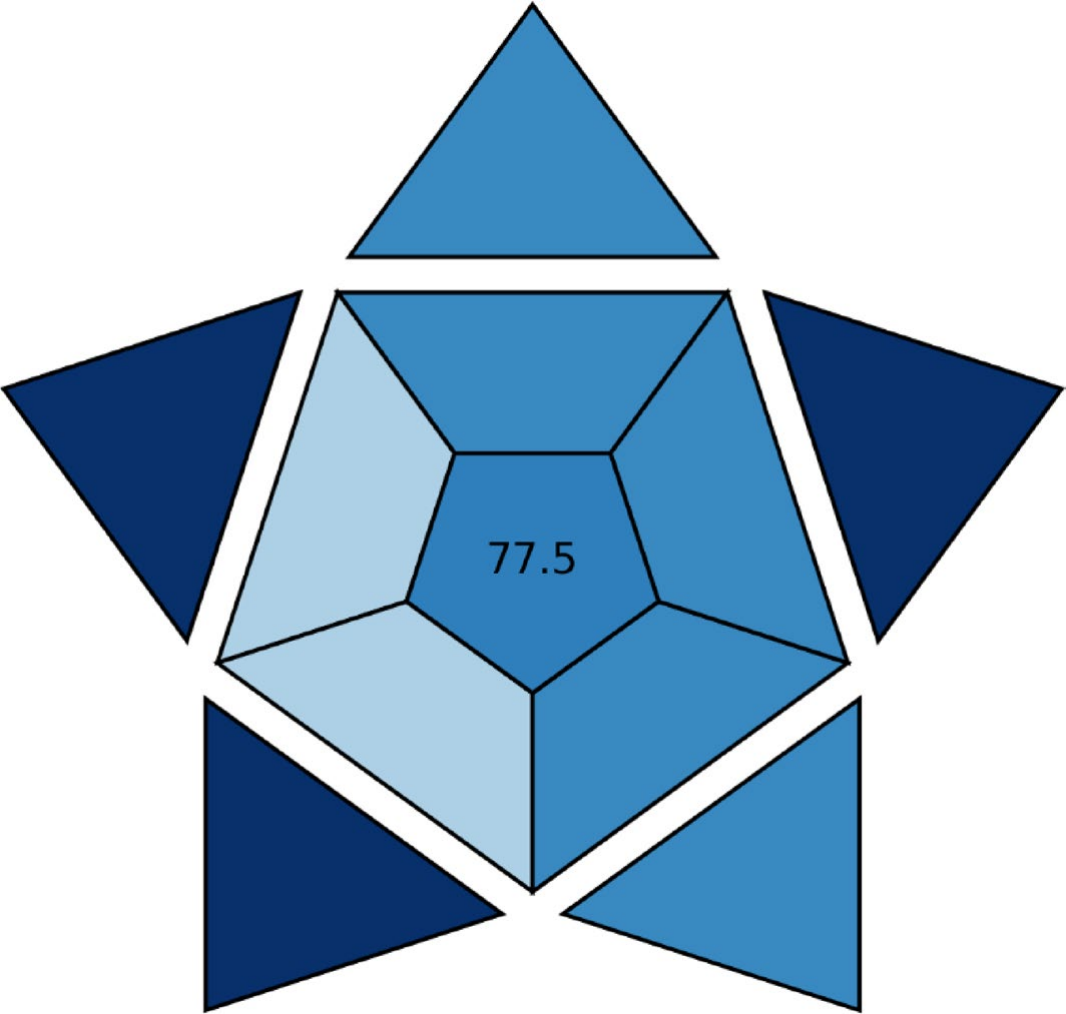
Blue Applicability Grade Index (BAGI)-colored pictogram of the developed LC-MS/MS method.

This tool employs a pictogram, a scoring system, and a color scale to represent the greenness of evaluated assays. The score range is 25 to 100 and a higher BAGI metric score indicates the assay is more environmentally friendly. Consequently, the developed method was given a 77.5 BAGI score, indicating its high practicality and applicability as presented in (Fig. 5).

#### Analytical greenness metric for sample Preparation (AGREEprep)

**Fig. 6:**
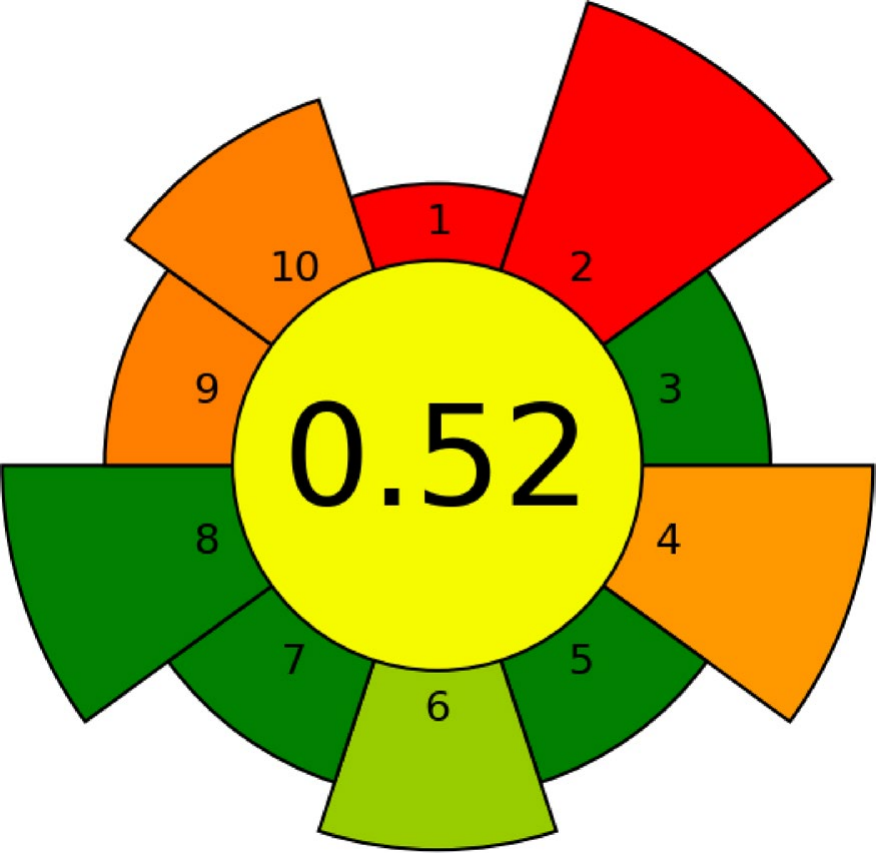
Analytical Greenness Metric for Sample Preparation (AGREEprep)-colored pictogram of the developed LC-MS/MS method.

The AGREEprep^28^ thoroughly assesses the greenness during sample preparation by integrating the evaluation procedure with the ten fundamental principles of ecologically friendly sample preparation, which are converted into a scale from 0 to 1. The AGREEprep pictogram depicts the ten principles as ten segments encircling the central field, where each segment’s colour can range from red (score 0) to dark green (score 1). The final score for the established LC-MS/MS method was 0.52 (Fig. 6).

#### Green analytical procedure index (GAPI)

**Fig. 7:**
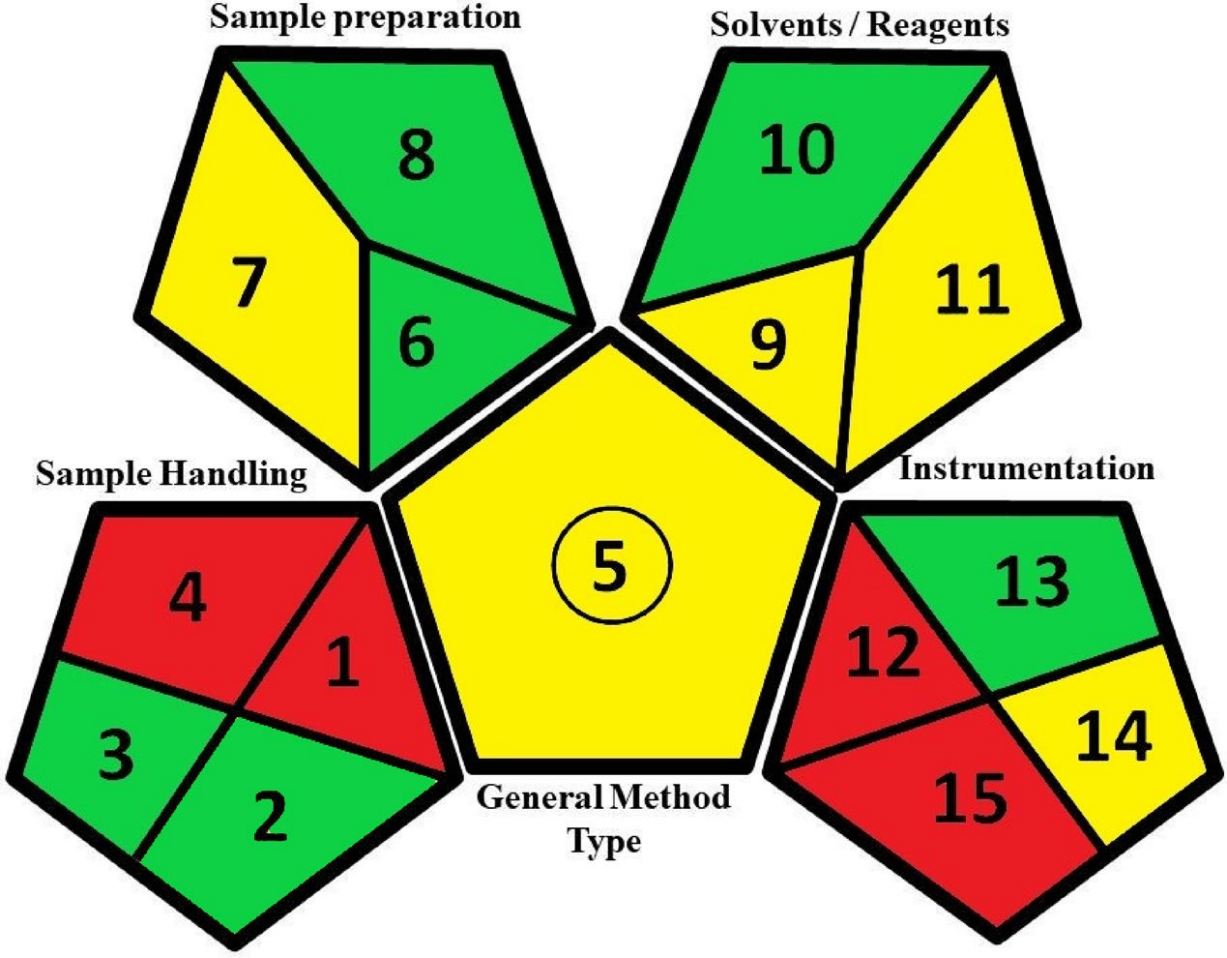
Green Analytical Procedure Index (GAPI)-colored pictogram of the developed LC-MS/MS method.

This is a method employed for the assessment of full procedural steps, categorized into five sections that delineate various factors within each segment^29^. The first part covers sample transportation, storage, collection, and preservation. The second aspect concerns the preparation of the sample. The third one pertains to the solvents and reagents used. The fourth aspect pertains to instrumentation, whereas the fifth concerns the general type of method. Based on the procedure’s safety and greenness, the GAPI tool is displayed as a pictogram of three colours in each section. The developed method exhibited six green aspects, five yellow aspects, and four red aspects (Fig. 7).

### Pharmacokinetic PK study

**Fig. 8:**
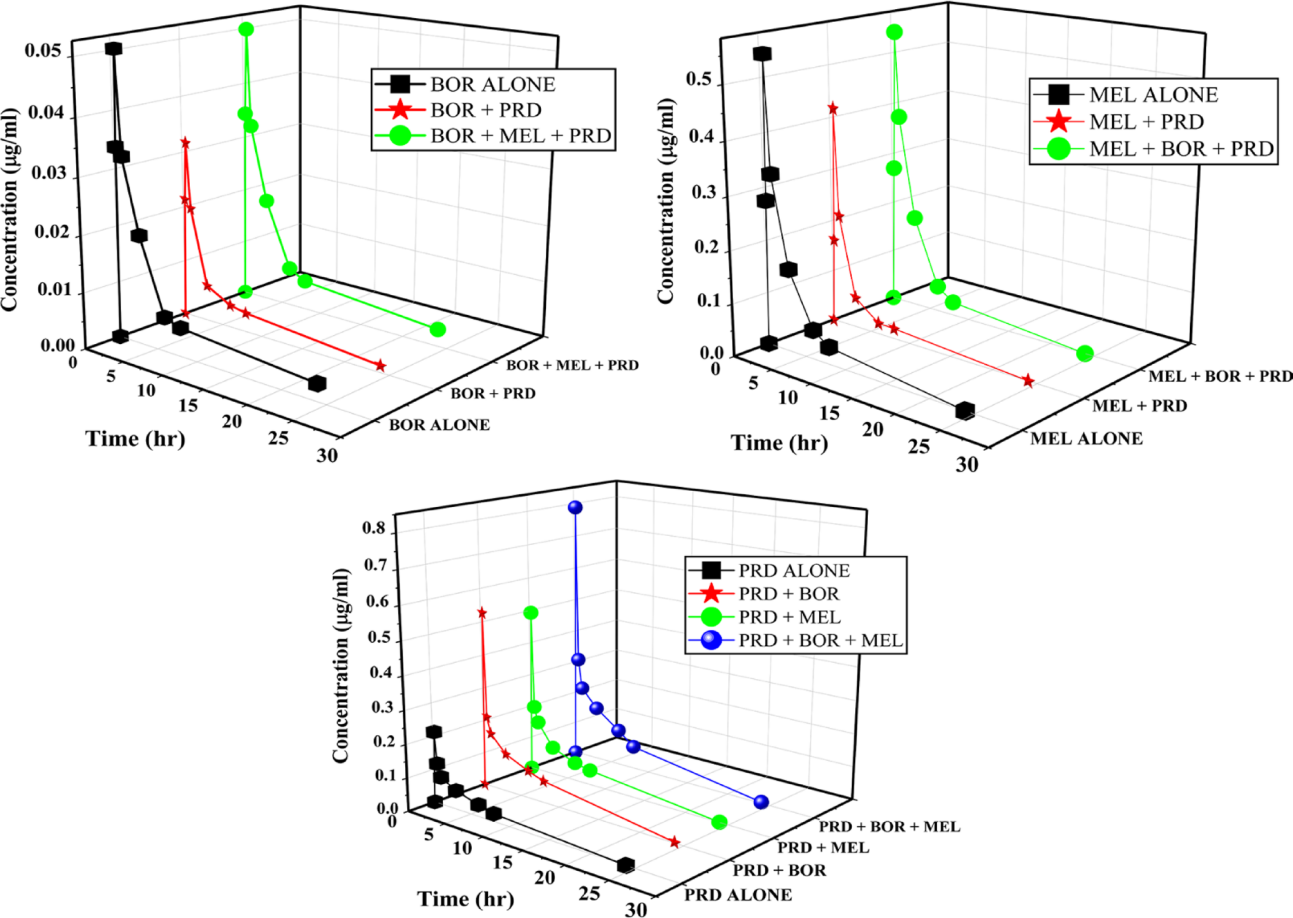
Graphs of plasma concentration versus time following the administration of BOR (0.25 mg/kg group 2), MEL (2 mg/kg group 3), PRD (10 mg/kg group 4) compared with their combination between BOR+PRD (group 5), MEL+PRD (group 6) and BOR+ MEL+PRD (group 7) by developed LC-MS/MS method.

Following treating rats with BOR, MEL, and PRD individually or in combination, the established LC-MS/MS technique was used to measure their concentration at different time intervals to estimate the PK parameters. The concentrations were obtained by using the regression equations previously computed and displayed with the corresponding relative time intervals (Fig. 8). The parameters listed in Table 6, terminal half-life (t_1/2_), time to C_max_ (t_max_), maximum observed plasma drug concentration (C_max_), elimination rate constant (K), drug clearance (CL), the volume of distribution (Vd), area under the plasma concentration–time curve (AUC) all and infinity, were calculated. In addition, PK parameters were analyzed using ANOVA and the Newman-Keuls Multiple Comparison Test Tables 7 and 8 to determine if there was a statistically significant difference between the groups in which each was administered single or in combination. The results showed visible dynamic shifts in the tested PK parameters when tri-therapy or di-therapy was administered together compared to monotherapy. A visible variation in the time of the observed peak plasma concentration and the maximum plasma concentration was shown. The AUC, which represents the overall amount of medication absorbed, varied as well. Remarkably, a combination of either MEL or BOR with PRD showed a decrease in C_max_ and AUC. Inversely, the PRD^’^s effect on MEL or BOR increased their K, Vd, and CL. Interestingly, the concentration of either BOR or MEL was restored when given concurrently in combination with PRD as tri-therapy and the restored concentration was not significantly different from the individual concentration.

**Table 6:** Pharmacokinetic parameters of bortezomib, melphalan, and prednisolone in rat plasma after intra-peritoneal administration of 0.25 mg/kg dose of bortezomib, 2 mg/kg dose of melphalan, and 10 mg/kg dose of prednisolone in single and combined dose.

Parameters*	BOR			MEL			PRD			
	alone	BOR + PRD	BOR + MEL + PRD	alone	MEL + PRD	MEL + BOR + PRD	alone	PRD + BOR	PRD + MEL	PRD + BOR + MEL
t_1/2_ (hr)	10.44	9.41	10.17	1.07	1.44	1.14	2.30	3.05	3.79	3.77
C _max_	0.0504	0.0318	0.0499	0.5436	0.4191	0.5416	0.2147	0.4946	0.5291	0.7894
T _max_ (hr)	0.5	0. 5	0.5	0.5	0.5	0.5	0.25	0.25	0.25	0.25
Elimination rate constant (K)	0.0664	0.0736	0.0681	0.6481	0.4812	0.6099	0.3014	0.2273	0.1827	0.1841
Volume of distribution (Vd) (mL)	15.26	26.97	14.74	2.05	4.96	2.12	52.06	38.23	35.59	28.01
Clearance (Cl) (mL/min)	1.01	1.99	1.01	1.33	2.39	1.29	15.69	8.69	6.50	5.16
AUC_0–t_ (µg/mL.h)	0.2012	0.1093	0.2043	1.51	0.8377	1.55	0.6370	1.15	1.52	1.92
AUC_0–∞_ (µg/mL.h)	0.2467	0.1259	0.2489	1.51	0.8377	1.55	0.6373	1.15	1.54	1.94

*Average of five rats.

**Table 7:** The ANOVA test compared Pharmacokinetic parameters between the single and combined groups.

Drugs	ANOVA	PK parameters				
		t _1/2_	K	Vd	Cl	AUC_0−t_
BOR	Between groups					
	SS	10.04	0.00043	430.31	3.39	0.0297
	df	2				
	MS	5.02	0.00021	215.16	1.70	0.0148
	F	8.23	9.34	180.27	161.26	286.85
	Within groups					
	SS	7.32	0.00028	14.32	0.1263	0.0006
	df	12				
	MS	0.6102	0.000023	1.19	0.0105	0.000052
	Total					
	SS	17.36	0.00071	444.64	3.52	0.0303
	dF	14				
MEL	Between groups					
	SS	0.4880	0.0987	24.33	2.95	1.37
	df	2				
	MS	0.2440	0.0494	12.16	1.47	0.6827
	F	15.58	12.51	264.69	26.34	12.19
	Within groups					
	SS	0.1880	0.0473	0.5514	0.6709	0.6719
	df	12				
	MS	0.0157	0.0039	0.0460	0.0559	0.0560
	Total					
	SS	0.6760	0.1461	24.88	3.62	2.04
	dF	14				
PRD	Between groups					
	SS	6.84	0.0453	1555.34	337.77	4.57
	df	3				
	MS	2.28	0.0151	518.45	112.59	1.52
	F	33.10	51.48	98.09	91.46	49.76
	Within groups					
	SS	1.10	0.0047	84.57	19.70	0.4896
	df	16				
	MS	0.0688	0.00029	5.29	1.23	0.0306
	Total					
	SS	7.94	0.0499	1639.91	357.47	5.06
	dF	19				

**Table 8:** The Newman–Keuls multiple comparison test to identify significant differences among groups.

Drugs	ANOVA	PK parameters				
		t _1/2_	K	Vd	Cl	AUC_0−t_
BOR	BOR alone vs. BOR + PRD	Yes	Yes	Yes	Yes	Yes
	BOR alone vs. BOR + MEL + PRD	No	No	No	No	No
	BOR + PRD vs. BOR + MEL + PRD	No	No	Yes	Yes	Yes
MEL	MEL alone vs. MEL + PRD	Yes	Yes	Yes	Yes	Yes
	MEL alone vs. MEL + BOR + PRD	No	No	No	No	No
	MEL + PRD vs. MEL + BOR + PRD	Yes	Yes	Yes	Yes	Yes
PRD	PRD alone vs. PRD + BOR	Yes	Yes	Yes	Yes	Yes
	PRD alone vs. PRD + MEL	Yes	Yes	Yes	Yes	Yes
	PRD alone vs. PRD + BOR + MEL	Yes	Yes	Yes	Yes	Yes
	PRD + BOR vs. PRD + MEL	Yes	Yes	No	No	Yes
	PRD + BOR vs. PRD + BOR + MEL	Yes	Yes	Yes	Yes	Yes
	PRD + MEL vs. PRD + BOR + MEL	No	No	Yes	No	Yes

Based on the current PK knowledge, it was visible that PRD led to more frequent responses in PK parameters upon its concomitant IP administration with BOR and/or MEL. From the above PK parameters, the variability in the plasma concentrations of MEL or BOR upon their dual or sole use with PRD in dual therapy may be due to the variations in the protein binding, absorption, distribution, or clearance of MEL or BOR. Also, we can hypothesize that single IP therapy with MEL or BOR or with a combination of three chemotherapeutic drugs can be capable of producing good responses, great exposure, and an expected long survival in MM patients by IP route. At all, a combination of the three agents in VMP may represent a more rational therapeutic approach for myeloma. Given these results, more clinical research is needed to guarantee precise results and reliable therapeutic drug monitoring or to increase awareness of the possibility of drug-drug interactions.

## Conclusion

The proposed LC-MS/MS method presents a novel way to quantify the co-administered medicines BOR, MEL, and PRD simultaneously in the (VMP) regimen. The purpose of this newly created, eco-friendly, and efficient analytical approach is to investigate the PK characteristics of the medications that build up the VMP combination, which is useful for MM patients. Rapid analysis with excellent sensitivity, precision, and repeatability was achieved by the developed LC-MS/MS method. To reduce the environmental impact and potential biohazards, every stage of the process has been carefully assessed in light of green analytical chemistry principles. The studied drugs were quantified in plasma to assess the effect of each monotherapy in the selected regimen, in addition to the concomitant administration shown by MP, VP, or VMP on their PKs after their IP administration into six separate groups of rats. Most PK parameters showed statistically significant differences between the groups under study, suggesting the potential for drug-drug interactions among the medications used in the regimen. The PK findings were consistent with the theory that multi-combinations of VMP can lead to more successful MM disease control. Based on the significance of the findings from the observed statistical PK data, we are going to do future clinical studies on the VMP regimen relying on our well-established LC-MS/MS method, which will offer an appropriate bioanalytical tool for tracking the therapeutic medications under study.

## Data Availability

All data generated or analyzed during this study are included in this published article.
